# Differentiated parietal connectivity of frontal regions for “what” and “where” memory

**DOI:** 10.1007/s00429-012-0476-4

**Published:** 2012-11-10

**Authors:** C. Rottschy, S. Caspers, C. Roski, K. Reetz, I. Dogan, J. B. Schulz, K. Zilles, A. R. Laird, P. T. Fox, S. B. Eickhoff

**Affiliations:** 1 Institute for Neuroscience and Medicine (INM-1, INM-2, INM-4), Research Center Jülich, Leo-Brandt Str. 5, 52425 Jülich, Germany; 2 Department of Neurology, RWTH Aachen University, Aachen, Germany; 3 Research Imaging Institute, University of Texas Health Science Center, San Antonio, TX, USA; 4 JARA-Translational Brain Medicine, Juelich, Germany; 5 C. and O. Vogt Institute for Brain Research, Heinrich-Heine-University Duesseldorf, Duesseldorf, Germany; 6 Institute of Clinical Neuroscience and Medical Psychology, Heinrich-Heine University, Düsseldorf, Germany; 7 Department of Psychiatry, Psychotherapy and Psychosomatics, RWTH Aachen University, Aachen, Germany

**Keywords:** Action, ALE, Cognition, Premotor, Resting state, Streams

## Abstract

In a previous meta-analysis across almost 200 neuroimaging experiments, working memory for object location showed significantly stronger convergence on the posterior superior frontal gyrus, whereas working memory for identity showed stronger convergence on the posterior inferior frontal gyrus (dorsal to, but overlapping with Brodmann’s area BA 44). As similar locations have been discussed as part of a dorsal frontal—superior parietal reach system and an inferior frontal grasp system, the aim of the present study was to test whether the regions of working-memory related “what” and “where” processing show a similar distinction in parietal connectivity. The regions that were found in the previous meta-analysis were used as seeds for functional connectivity analyses using task-based meta-analytic connectivity modelling and task-independent resting state correlations. While the ventral seed showed significantly stronger connectivity with the bilateral intraparietal sulcus (IPS), the dorsal seed showed stronger connectivity with the bilateral posterior inferior parietal and the medial superior parietal lobule. The observed connections of regions involved in memory for object location and identity thus clearly demonstrate a distinction into separate pathways that resemble the parietal connectivity patterns of the dorsal and ventral premotor cortex in non-human primates and humans. It may hence be speculated that memory for a particular location and reaching towards it as well as object memory and finger positioning for manipulation may rely on shared neural systems. Moreover, the ensuing regions, in turn, featured differential connectivity with the bilateral ventral and dorsal extrastriate cortex, suggesting largely segregated bilateral connectivity pathways from the dorsal visual cortex via the superior and inferior parietal lobules to the dorsal posterior frontal cortex and from the ventral visual cortex via the IPS to the ventral posterior frontal cortex that may underlie action and cognition.

## Introduction

Concepts on the organisation of human memory are mainly based on the long-held dichotomy of short-term (STM), respectively, working memory (WM) and long-term memory (LTM) (Atkinson and Shiffrin 1968; Brown 1958; Hebb 1949; Peterson and Peterson 1959). Throughout the neuropsychological and neuroimaging literature, two complementary organizational aspects of the human WM system are consistently acknowledged. One is the presence of a “central executive” forming a capacity engaged in encoding, recall and manipulation of WM content independent of a specific modality (‘amodal processor’). The other aspect is a striking modularity of WM with differentiable function and neural correlates of various aspects such as verbal or non-verbal WM or remembering spatial (object location) versus feature-based (object identity) properties (Ragland et al. 2004; Shen et al. 1999; Thomason et al. 2009; Veltman et al. 2003, 2005). The latter distinction (object location vs. features) is particularly interesting, as it relates to a broad distinction between “what” and “where” pathways, which have been described in several functional systems. The idea of two different pathways or streams (what and where) has been discussed in various functional systems in the brain but is possibly best known and widely acknowledged in the visual system. Here, ventral aspects of the occipital and adjacent inferior temporal cortex, in particular along the fusiform gyrus, have consistently been shown to respond preferentially or even exclusively to objects, faces and similar shapes such as letters. In contrast, the dorsal occipital and superior parietal cortex are more preferentially engaged during visual-spatial tasks (Milner and Goodale 2008; Ungerleider et al. 1998; Ungerleider and Haxby 1994). Furthermore, Ungerleider and colleagues were among the first to present evidence for an extension of these functional preferences into the frontal lobe, prompting the notion of dorsal and ventral pathways or streams. While most prominent in the visual system, the concept of discernible streams devoted to the processing of “what” and “where” features has likewise been proposed in the auditory system. In particular, there is solid evidence for separate processing of spatial and non-spatial information along different pathways in non-human primates (Rauschecker and Tian 2000), which seems to be also present in the human auditory system (Hafke 2008; Krumbholz et al. 2007; Loui et al. 2008). Converging, these findings suggest a predominant processing of non-spatial auditory features (such as fundamental frequency or pitch -envelopes) in a ventral auditory stream including inferior frontal and temporal areas, while spatial features such as binaural time- and amplitude differences seem to be processed mainly in a dorsal stream including the superior frontal sulcus and inferior parietal lobe (Arnott et al. 2004). In addition to these streams of visual and auditory sensory processing, the idea of pathways has also been discussed in the context of language processing. A ventral language stream connecting the middle temporal lobe and the ventrolateral prefrontal cortex has been proposed to map sound to meaning, while a dorsal stream, connecting superior temporal lobe and premotor cortices seems to be more involved in converting sound to articulation (Rauschecker 2012; Saur et al. 2008). Finally, there is also evidence from invasive tracer and electrophysiological studies in non-human primates for dorsal and ventral pathways between parietal and premotor areas (Luppino et al. 1999; Matelli et al. 1998). In particular, dorsal premotor regions seem to connect predominantly to the superior parietal cortex and subserve reaching in space while more ventral aspects of the premotor cortex interact more strongly with the intraparietal sulcus for object manipulation, a finding that has more recently been corroborated in humans using diffusion tractography (Tomassini et al. 2007). In summary, a large body of evidence thus points to the existence of “dorsal” and “ventral” streams in the brains of humans and non-human primates that are preferentially dedicated to the spatial and non-spatial, object related processing, respectively. That is, the distinction between dorsal (“where”) pathways processing spatial codes and relationships as well as accounting for different coordinate systems such as egocentric (eye or body related) and allocentric representations on one hand and ventral (“what”) pathways dealing with non-spatial properties of objects such as its shape and colours, arrangement of local elements as well as, potentially, semantic associations on the other seems to represent a fundamental organizational principle of the primate brain.

In a recent coordinate-based meta-analysis of neuroimaging studies, we could demonstrate a similar distinction in the context of working memory (and hence not motor-related) tasks. In particular, there was a clear distinction between brain regions that are reliably activated in tasks requiring the subjects to remember object identity and object location, respectively (Rottschy et al. 2012). While memory for object identity as compared to object location was significantly more likely to recruit the bilateral posterior inferior frontal gyrus (dorsal to, but overlapping with area 44), significantly stronger convergence in tasks requiring to memorize object location, as compared to identity, was found bilaterally on the posterior superior frontal gyrus and the adjacent precentral gyrus. That is, we found a consistent (across paradigms and experiments) distinction between the neuronal correlates of presumed “what” and “where” aspects of working memory in the posterior frontal cortex. Interestingly, the locations of these segregated representations of “what” and “where” memory moreover closely resemble the distinction between ventral and dorsal premotor areas in humans and other primates (Geyer et al. 1999; Rizzolatti and Luppino 2001); (Schubotz and von Cramon 2003; Tomassini et al. 2007). It is important to empathize, however, that in spite of this topographic similarity, our seeds reflect differential activation of the frontal cortex in relation to spatial and object-centred working memory processes and can therefore not be assumed to necessarily correspond to the dorsal and ventral premotor cortex, respectively. The aim of the present study was to delineate the functional connectivity of these regions for “what” and “where” aspects of working memory in human posterior frontal cortex. Moreover, we aim at investigating whether functional connectivity analyses may delineate more generalized streams of “what” and “where” pathways extending into the visual system based on these frontal, WM-related seeds.

## Materials and methods

### Coordinate-based meta-analysis: seeds

**Fig. 1:**
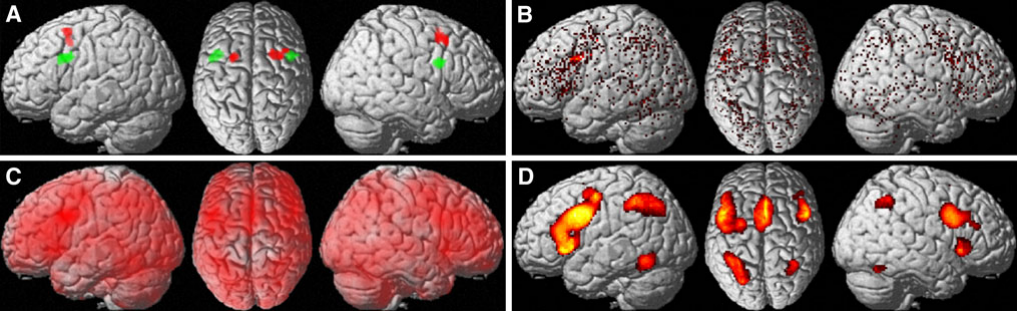
**a** Segregation of the frontal cortex as revealed by a coordinate-based meta-analysis of working memory studies (Rottschy et al. 2012). Regions where experiments on object location showed a significantly higher convergence of reported activations than those probing memory for object identity are shown in *red*. Regions showing stronger convergence of activation in experiments on object identity are displayed in *green*. The left ventral region serves as exemplary seed to illustrate the meta-analytic connectivity modelling (MACM) approach in **b**–**d**. **b** Activation foci of all experiments in the BrainMap Database, which show at least one activation in the seed region (left posterior inferior frontal gyrus). **c** The reported coordinates, which are shown in **b** are treated as probability distributions, which indicate that the “true” locations are modelled as 3D Gaussians. **d** Random effect inference against a null-distribution of random spatial association across studies

The current study draws on the results of a previous coordinate-based meta-analysis across functional magnetic resonance imaging studies on working memory. This meta-analysis revealed a clear distinction in the neural correlates of working memory for object location and object identity (Rottschy et al. 2012). Whereas the former (“where”) showed significantly stronger convergence on the bilateral posterior superior frontal gyrus (MNI peak coordinates: left −20/10/56; right 24/12/15), the latter (“what”) showed stronger convergence more ventrally on the posterior inferior frontal gyrus [dorsal to Brodmann’s area BA 44; and only marginally overlapping with this area (2.6 % of the left and 4.9 % of the right seed were located in BA 44)]; MNI peak coordinates: left −40/12/32; right 42/6/26, cf. Fig. 1a). The regions observed in this meta-analysis, were taken as seed regions for further analysis of task-dependent (MACM) and task-independent (resting state) functional connectivity (Eickhoff and Grefkes 2011).

### Meta-analytic connectivity modelling

Functional connectivity of the seeds during task performance was delineated by meta-analytic connectivity modelling (MACM). This approach to functional connectivity assesses which brain regions are co-activated above chance with a particular seed region in functional neuroimaging experiments. This method takes advantage of the fact that functional imaging studies are normally presented in a highly standardized format using ubiquitously employed standard coordinate systems, and the emergence of large-scale databases, which store these information. The first step in MACM is to identify all these experiments in a database that activate the seed region. After that, quantitative meta-analysis is employed to test for convergence across the foci reported in these experiments. As experiments are selected by activation in the seed, the highest convergence will be observed in the seed region. Significant convergence of reported foci in other brain regions, however, indicates consistent co-activation, i.e., functional connectivity with the seed (Eickhoff et al. 2010; Robinson et al. 2010).

In this study we employed the BrainMap database (Laird et al. 2009a, 2011a) (http://www.brainmap.org), which contained at the time of analysis approximately 10,000 neuroimaging experiments. We only included studies that reported group analyses of functional mapping experiments of healthy subjects. Those studies that dealt with disease or drug effects were excluded. No further constraints (e.g., on acquisition and analysis details, experimental design, or stimulation procedures) were enforced, yielding approximately 6,500 experiments for analysis. Note that we considered all eligible BrainMap experiments because any pre-selection of taxonomic categories would have constituted a fairly strong a priori hypothesis about how brain networks are organized. This was a conservative approach, given that an understanding of how psychological constructs, such as action and cognition, map on regional brain responses remains elusive (Laird et al. 2009a; Poldrack 2006, 2011). In particular, using broad behavioural domains like “action” would add only moderately to the specificity of the obtained results given the large heterogeneity of “action-related” experiments. In turn, more specific domain- or paradigm-filtering (only action imitation, only go/no-go tasks) would, however, introduce to many a priori constraints. Given the assumption that the seed regions in the posterior frontal cortex may be engaged by action- and cognition-centred experiments, we opted for a completely data-driven approach in which experiments were only selected based on the location of their activations.

To delineate task-based functional connectivity, i.e., co-activations of the regions implied by the previous meta-analysis (dorsal superior and inferior frontal gyrus) (Fig. 1a), we thus first identified all experiments in the BrainMap database that reported group analyses of functional mapping experiments of healthy subjects and featured at least one focus of activation in the respective seed (Fig. 1b). Subsequently, the convergence of foci reported in these experiments was quantified using the revised activation likelihood estimation (ALE) algorithm (Eickhoff et al. 2010) for coordinate-based meta-analysis of neuroimaging results (Eickhoff et al. 2009; Laird et al. 2009a, b; Turkeltaub et al. 2002) implemented as in-house MATLAB tools. This algorithm aims to identify areas showing a convergence of reported coordinates across experiments, which is higher than expected under a random spatial association. The key idea behind ALE is to treat the reported foci not as single points, but rather as centres for 3D Gaussian probability distributions capturing the spatial uncertainty associated with each focus (Fig. 1c). The probabilities of all foci reported in a given experiment were then combined for each voxel, resulting in a modelled activation (MA) map (Eickhoff et al. 2012; Turkeltaub et al. 2012). Taking the union across these MA maps yielded voxelwise ALE scores describing the convergence of results at each particular location of the brain. To distinguish ‘true’ convergence between studies from random convergence (i.e., noise), ALE scores were compared to an empirical null-distribution (Eickhoff et al. 2012) reflecting a random spatial association between experiments (Fig. 1d). Hereby, a random-effects inference is invoked, focussing on inference on the above-chance convergence between studies, not clustering of foci within a particular study. The *p* value of a “true” ALE was then given by the proportion of equal or higher values obtained under the null-distribution. The resulting non-parametric *p* values for each meta-analysis were then thresholded at a cluster-level corrected threshold of *p* < 0.05 (cluster-forming threshold at voxel level *p* < 0.001) and transformed into *Z* scores for display.

Difference maps comparing task-based functional connectivity maps of the ventral (what) and dorsal (where) seeds were established by first calculating the voxelwise differences of the *Z* scores obtained from the inspected MACM maps. The experiments contributing to either analysis were then pooled and randomly divided into two groups of the same size as the sets of contrasted experiments (Eickhoff et al. 2011). Voxelwise ALE scores for these two randomly assembled groups were subtracted from each other and recorded. Repeating this process, 10,000 times yielded an empirical null distribution of ALE-score differences between the two conditions. Based on this permutation procedure, the map of true differences was then thresholded at a posterior probability of *p* > 0.95 for a true difference between the two samples. The resulting maps were then masked with the respective main effect of the minuend connectivity map to avoid obtaining significant connectivity in voxels of the difference map that do not show significant co-activation on the underlying connectivity map. Furthermore, only regions with at least 20 cohesive voxels were considered in the resulting difference maps.

### Task independent “resting state” connectivity modelling

Resting state fMRI images of 100 healthy volunteers without records of neurological or psychiatric disorders were acquired. All subjects gave written informed consent to the study protocol, which had been approved by the local ethics committee of the University of Bonn as the data were acquired as part of an independent collaborative project. During the resting state scans subjects were instructed to keep their eyes closed and to think about nothing in particular but not to fall asleep (which was confirmed by post-scan debriefing). For each subject, 300 resting state EPI images were acquired using blood-oxygen-level-dependent (BOLD) contrast [gradient-echo EPI pulse sequence, TR = 2.2 s, TE = 30 ms, flip angle = 90°, in plane resolution = 3.1 × 3.1 mm^2^, 36 axial slices (3.1 mm thickness) covering the entire brain]. The first four scans were excluded from further processing analysis using SPM8 (http://www.fil.ion.ucl.ac.uk/spm). The EPI images were first corrected for movement artefacts by affine registration using a two pass procedure in which the images were first aligned to the initial volumes and subsequently to the mean after the first pass. The obtained mean EPI of each subject was then spatially normalized to the MNI single subject template (Holmes et al. 1998) using the ‘unified segmentation’ approach (Ashburner and Friston 2005). The ensuing deformation was applied to the individual EPI volumes. At last, images were smoothed by a 5-mm FWHM Gaussian to improve signal-to-noise ratio and compensate for residual anatomical variations.

**Fig. 2:**
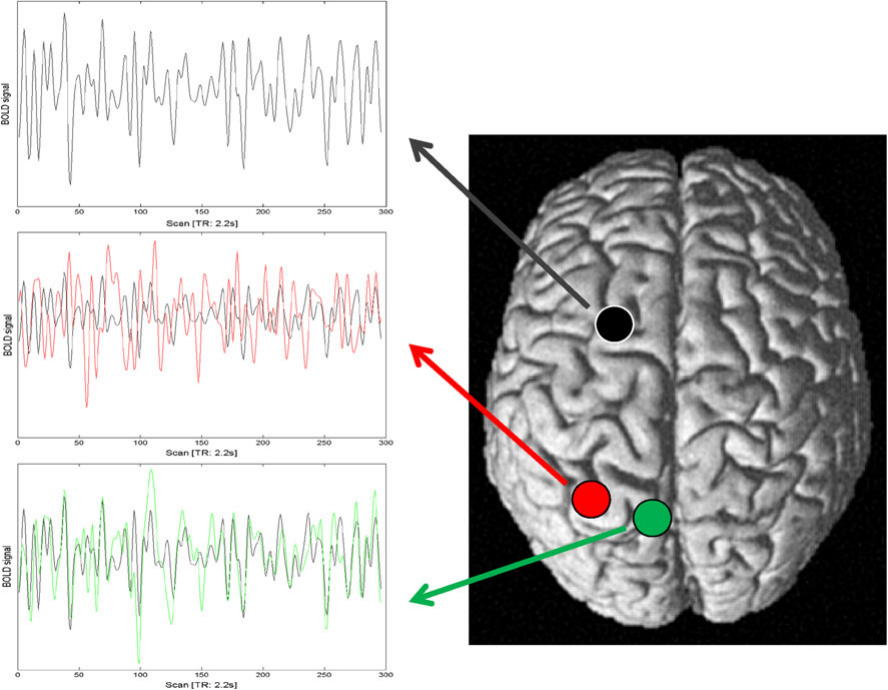
Time series of the dorsal seed region (posterior superior frontal gyrus) in a single subject is shown in *grey*. From the same subject, the time series of an uncorrelated voxel is shown in *red* and that of a correlated voxel in *green*

The time-series data of each voxel were processed (Fig. 2) as follows (Eickhoff et al. 2011; Weissenbacher et al. 2009; Zu Eulenburg et al. 2012). In order to reduce spurious correlations, variance that could be explained by the following nuisance variables was removed: (1) the six motion parameters derived from the image realignment, (2) the first derivative of the realignment parameters, (3) mean grey matter, white matter and CSF signal per time-point as obtained by averaging across voxels attributed to the respective tissue class in the SPM 8 segmentation, and (4) coherent signal changes across the whole brain as reflected by the first five components of a principal component analysis (PCA) decomposition of the whole-brain time-series (PrinCor denoising). All nuisance variables entered the model as first and all but the PCA components also as second order terms as previously described by Behzadi et al. (2007) and shown by Chai et al. (2012) to increase specificity and sensitivity of the analyses. Data were then band pass filtered preserving frequencies between 0.01 and 0.08 Hz, since meaningful resting state correlations will predominantly be found in these frequencies given that the bold-response acts as a low-pass filter (Biswal et al. 1995; Fox and Raichle 2007).

We again used the same seed regions as for the MACM analysis, i.e., the clusters of differential activity for “what” and “where” memory as obtained from the meta-analysis (Rottschy et al. 2012). Time courses were extracted for all voxels within the particular cluster and expressed as their first eigenvariate. Linear (Pearson) correlation coefficients between the time series of the seed regions and all other grey matter voxels in the brain were computed to quantify resting-state functional connectivity. These voxelwise correlation coefficients were then transformed into Fisher‘s *Z* scores and tested for consistency across subjects in a random-effects analysis. In particular, the Fisher’s *Z* transformed whole-brain connectivity maps of all seeds were included in an ANOVA model accounting for non-sphericity in the data originating from the fact that the different seeds represented correlated measures within each subject and unequal variance between seeds and subjects. Appropriate linear contrasts were then applied to test for regions strongly connected to the seed on the posterior inferior and posterior superior frontal gyrus, respectively. The results of this random-effects analysis were then thresholded at a cluster-level corrected threshold of *p* < 0.05 (cluster-forming threshold: *p* < 0.001 at voxel level).

### Cross-validation of MACM and resting state

To detect areas showing task-dependent and task-independent functional connectivity with the seed regions obtained from a meta-analysis, we performed a conjunction analysis between MACM and resting state analyses using the minimum statistics (Jakobs et al. 2012; Nichols et al. 2005). We aimed at identifying voxels that showed significant functional connectivity with the seed in the analysis of interactions in both task-dependent and task-independent state. We therefore delineated such consistent connectivity by computing the intersection of the (cluster-level FWE corrected) connectivity maps from the two analyses. The main focus of our work was on the conjunction of differences. We wanted to identify regions, which showed significantly stronger coupling with, e.g., the ventral as compared to the dorsal seeds in the analysis of task-based and task-independent functional connectivity. We thus additionally computed the conjunction (across modalities) of the contrasts (between seeds). That is to identify regions significantly stronger connected to the ventral (what) as compared to the dorsal (where) seed in both task-dependent and task-independent functional connectivity. We computed the intersection between regions showing significant effects for “connectivity with the ventral > connectivity with the dorsal seed” in the MACM analysis and regions showing significant effects for “connectivity with the ventral > connectivity with the dorsal seed” in the resting-state analysis.

All results were anatomically labelled by reference to probabilistic cytoarchitectonic maps of the human brain using the SPM Anatomy Toolbox (Eickhoff et al. 2005, 2006, 2007a). Using a maximum probability map (MPM), activations were assigned to the most probable histological area at their respective locations. Details on these cytoarchitectonic regions are found in the following publications reporting on Broca’s region (Amunts et al. 1999), inferior parietal cortex (Caspers et al. 2006, 2008), as well as superior parietal cortex and intraparietal sulcus (Choi et al. 2006; Scheperjans et al. 2008a, b). Regions, which are not yet cytoarchitectonically mapped based on observer-independent histological examination, were labelled macroanatomically by the probabilistic Harvard–Oxford cortical structural atlas, rather than providing tentative histological labels based on volume approximations of the (schematic) Brodmann atlas.

### Functional characterization analysis

The functional characterization of the clusters was based on the BrainMap meta-data that describe the classes of mental processes isolated by the archived experiments’ statistical contrasts. Behavioural domains comprise the main categories cognition, action, perception, emotion, and interoception, as well as their related sub-categories and denote the mental processes isolated by the respective contrast. In turn, paradigm classes categorize the specific task employed (see http://www.brainmap.org/scribe/ for the complete BrainMap taxonomy). For the functional characterization of the difference between the seeds or their ensuing networks, we proceeded as follows: first, we identified all experiments in the BrainMap database, which featured at least one focus of activation within one of the seeds/within the seed and its connected regions. That is, for the functional comparison between the posterior superior and posterior inferior frontal cortex, we identified all experiments activating within either of these regions. For the functional comparison between the networks connected to these seeds, we identified all experiments activating either (1) the posterior inferior frontal cortex and simultaneously one of the regions it is connected with or (2) the posterior superior frontal cortex and simultaneously one of the regions it is connected with. From this pool of experiments, the baserate is the a priori probability of any focus to lie in either of the two compared regions/networks (i.e., when randomly drawing a focus that activates superior inferior or superior posterior frontal cortex, what is the probability that it is the posterior one). The conditional probability of observing activity in a brain region given knowledge of the psychological process was then computed and compared to this baserate by means of a binomial test (*p* < 0.05, corrected for multiple comparisons using Bonferroni’s method). This allowed to characterize the functional profile of a cluster or network by identifying taxonomic labels, for which the probability of finding activation in the respective cluster was significantly higher given the respective label as compared to baseline.

## Results

### Contrasts of “what” and “where” memory in meta-analytic connectivity modelling

**Fig. 3:**
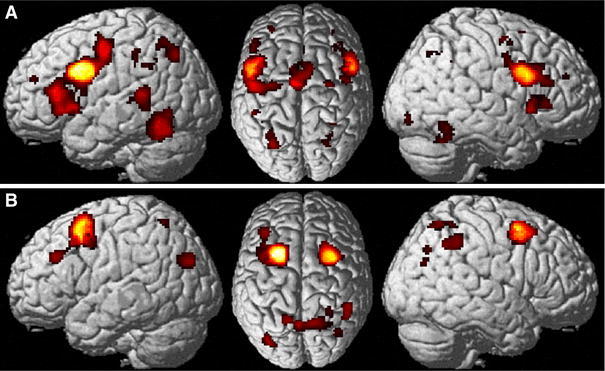
**a** Regions showing significantly stronger task-based (MACM) functional connectivity with the ventral as opposed to the dorsal posterior frontal seed. **b** Regions showing significantly stronger task-based (MACM) functional connectivity with the dorsal as opposed to the ventral posterior frontal seed

Significantly stronger connectivity with the ventral seeds, located on the posterior inferior frontal gyrus bilaterally, was found locally in Broca’s area and the caudal part of the LPFC (dorsolateral prefrontal cortex) as well as bilaterally in the cerebellar lobule VI (Diedrichsen et al. 2010), the basal ganglia (particularly the putamen) the ventral extrastriate cortex on the fusiform gyrus, (pre-) SMA, the intraparietal sulcus (hIP1 and hIP3) and the regions of the thalamus connected to the prefrontal cortex (Behrens et al. 2003). In the right hemisphere, stronger connectivity with the anterior insula, the middle cingulate cortex and the cerebellum (Lobule VIIa and the Vermis) was additionally found, while in the left hemisphere we found stronger connectivity with the middle temporal gyrus (Fig. 3a).

The dorsal seeds, located on the posterior superior frontal gyrus, in turn, showed stronger task-based functional connectivity with the superior parietal lobe (areas 7A and 7PC) and the inferior parietal cortex (areas PFm and PGa on the right hemisphere; area PGp on the left hemisphere) bilaterally (Fig. 3b).

### Contrasts of “what” and “where” memory in resting-state connectivity modelling

**Fig. 4:**
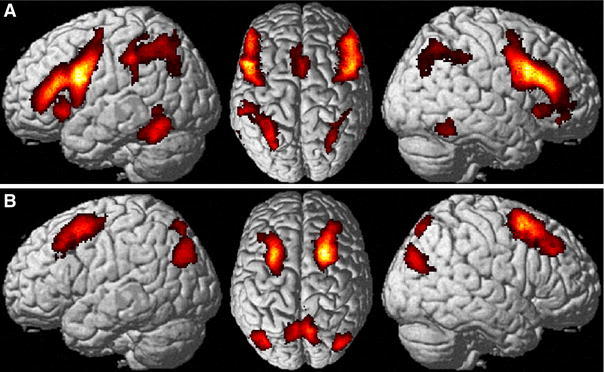
**a** Brain regions showing significantly stronger task independent (resting state) connectivity with the ventral as opposed to the dorsal frontal seed. Please note, that the parietal activation was predominantly located in the IPS but projects to the IPL in the lateral view. **b** Brain regions showing significantly stronger task independent (resting state) connectivity with the dorsal as opposed to the ventral seed region

In the task-independent resting-state functional connectivity analysis, the ventral seeds featured stronger connectivity bilaterally with Broca’s region, caudal LPFC, (pre-) SMA and adjacent the middle cingulate cortex, the basal ganglia and the ventral extrastriate cortex (fusiform gyrus) as well as the anterior intraparietal sulcus (hIP1-3) and adjacent inferior and superior parietal lobules bilaterally. On the right hemisphere, stronger connectivity was additionally found in the anterior insula (Fig. 4a).

The dorsal seeds, in turn, showed stronger connectivity with the bilateral superior parietal lobe/precuneus (areas 7A and 7P) and posterior inferior parietal cortex (PGp) (Fig. 4b).

### Conjunction analysis across both approaches

As demonstrated above, both approaches (task-based MACM and task-independent resting state functional connectivity) revealed similar regions, which showed stronger connectivity with the ventral and dorsal seed, respectively. The conjunction analysis across both approaches, hence demonstrated bilateral significantly stronger task-based and task-independent functional connectivity of the ventral seeds with Broca’s region (BA 44, BA 45), anterior insula, caudal part of the LPFC, (pre-) SMA, intraparietal sulcus (hIP1-3), and extrastriate visual cortex as well as the basal ganglia.

**Fig. 5:**
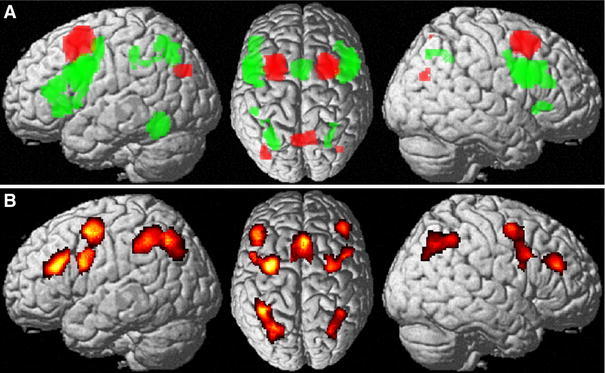
**a** Conjunction across task dependent (MACM) and task independent contrast analyses. Regions, which showed stronger connectivity with the posterior inferior frontal cortex are shown in* green*, while those regions, which showed stronger connectivity with the posterior superior frontal cortex are shown in *red*. **b** Conjunction across both approaches [task dependent (MACM) and independent (resting state) functional connectivity] and seeds (ventral and dorsal posterior inferior and posterior superior frontal cortex)

In contrast, the posterior superior frontal gyrus (dorsal seed) bilaterally showed significantly stronger connectivity across both approaches with the superior parietal lobe (areas 7A and 7P) and the posterior inferior parietal cortex (area PGp) bilaterally (Fig. 5a). These findings thus revealed several regions that show differential functional connectivity with the posterior inferior and posterior superior frontal cortex, respectively, across two fundamentally different states, i.e., during the performance of externally structured tasks and during a task-free resting state.

Furthermore, a conjunction analysis across both the bilateral dorsal and ventral seeds as well as over both approaches indicated that Broca’s region, caudal LPFC, intraparietal sulcus (areas hIP1-3), inferior parietal cortex (area PFm), and superior parietal lobe (area 7A) showed consistent connectivity with both seed regions across both states (Fig. 5b).

### Functional connectivity of parietal regions

In a follow-up analysis, we then assessed connectivity of the parietal regions showing differential connectivity with the ventral (posterior inferior frontal gyrus) and dorsal (posterior superior frontal gyrus) seed, i.e., the IPS and the SPL/IPC, respectively, using exactly the same approach as described above. This analysis served two purposes: first, we aimed to cross-validate our results by assessing if these regions would show in turn significant differences in their connectivity with the frontal seeds. Second, we aimed at potentially extending the delineated pathways further posterior into the visual domain, given that parietal regions may act as a link from visual to frontal regions.

In this supplementary analysis, we found that the SPL and posterior IPC showed significantly stronger task-based and task-independent functional connectivity with the superior parieto-occipital cortex [SPOC, a region, which has been argued to functionally correspond to the parietal reach region (PRR)] and dorsal extrastriate visual areas (V3d and the cortex in the vicinity of V5) as well as with the posterior superior frontal gyrus.

**Fig. 6:**
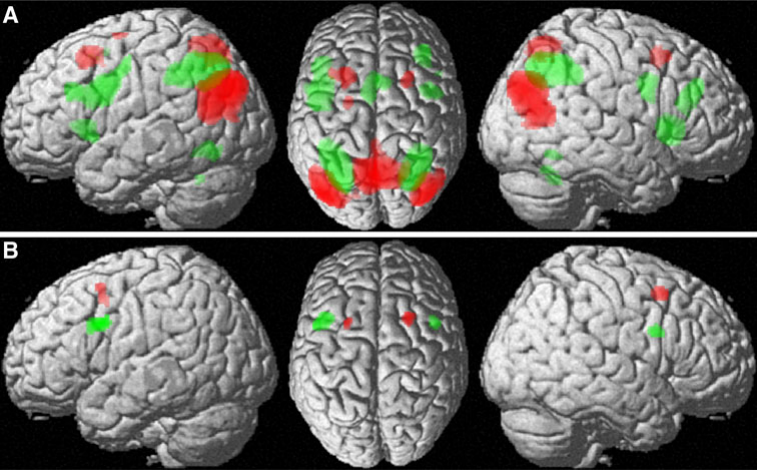
**a** Conjunction across task dependent (MACM) and task independent (resting state) connectivity differences between parietal regions, which showed connectivity with the dorsal and ventral frontal seed in the above shown analysis (cf. Fig. 5a). Regions, which showed stronger connectivity with the IPS are shown in *green*, while those regions, which showed stronger connectivity with the SPL/IPL are shown in *red*. **b** Significant differences in connectivity between the IPS and SPL/IPL in the posterior frontal cortex. Here it is shown, that IPS showed stronger connectivity with the bilateral ventral seeds and SPL/IPL showed stronger connectivity with the bilateral dorsal seeds. Convergence with the original seeds was ensured using these as a mask to the results shown in **a**

In contrast, the IPS, i.e., the parietal region more closely connected to the ventral seed, featured significantly stronger task-based and task-independent functional connectivity with bilateral ventral extrastriate visual cortex, the anterior insula, (pre-) SMA, and the posterior inferior frontal gyrus. Moreover, significant differences in connectivity with the right posterior lateral prefrontal cortex (LPFC) and the right cerebellum (Lobule VI) were also found (Fig. 6a).

Finally, we calculated the same MACM and resting state functional connectivity differences for the parietal regions showing differential connectivity with the two frontal seed regions (IPS vs. SPL/IPL) using the very same frontal seed regions as inclusive masks. The procedure revealed that, indeed, the significant distinction in connectivity patterns was reciprocal. That is, those regions that were defined by stronger connectivity with the ventral frontal seed also featured significantly stronger connectivity with this region when used as seeds themselves. In turn, regions that were defined by stronger connectivity with the dorsal seed also showed significantly stronger connectivity with this region (Fig. 6b). While this analysis is fundamentally circular in nature, the fact that significant effects could be seen for this “reverse” analysis in the original seeds attests to the robustness of the delineated fronto-parietal connectivity differences in spite of the inevitable, presumably method (MACM, resting-state)-specific noise in functional connectivity analysis.

### Functional characterization analysis

**Fig. 7:**
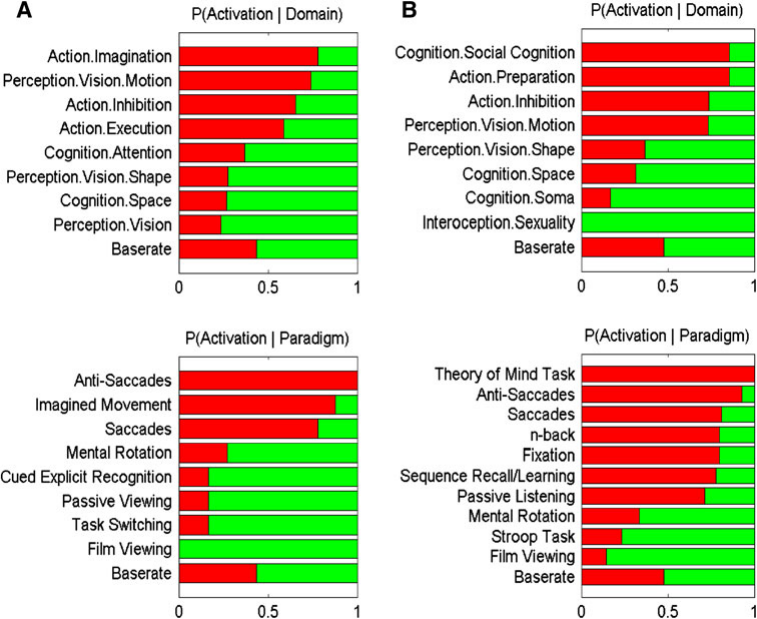
**a** Characterization of the functional differences between the networks formed by ventral (*green*) and dorsal (*red*) posterior frontal regions and their respective parietal connections. Behavioural domains are shown on *top*, paradigm classes on the *bottom*. **b** Characterization of the functional differences between the ventral (*green*) and dorsal (*red*) posterior (seed) frontal regions

As described before, the frontal seed regions show stronger connectivity with distinct parietal regions, respectively. The functional characterization of the networks found by these seeds and their respective parietal connections is shown in Fig. 7. Behavioural domains and paradigm classes significantly overrepresented in the dorsal seed and its associated network (IPL and SPL) were related to action (action inhibition, action imagination, action execution) and spatial tasks like anti-saccades, saccades and imagined movements (Fig. 7a). When considering only the seed region, a likewise preponderance for spatial- /action-related behavioural domains and paradigms came up (Fig. 7b). In addition, there was a significant association with social cognition, in particular theory of mind tasks, while this may seem surprising at first, it may well be explained by the high prevalence of “perspective taking” paradigms among these, which we require to shift the frame of spatial reference to the perspective of another person.

In contrast, the ventral seeds and the IPS featured a significant overrepresentation of behavioural domains related to visual shape and configuration (Perception.Vision.Space, these mainly being attributable to mental rotation tasks) processing as well as those related to general visual/attentive functions. The paradigm class analysis then revealed that the latter could be attributed to tasks involving passive and active (recognition) viewing of images as well as following film presentations (Fig. 7a). When considering only the seed region in the ventral posterior frontal cortex a very similar pattern emerges while additionally we found evidence for involvement in somatosensory and more general bodily perception (Fig. 7b).

## Discussion

### Summary of findings

In a previous coordinate-based meta-analysis of functional neuroimaging studies (Rottschy et al. 2012), we found consistent differences between working memory tasks requiring the subjects to memorize object identity compared to those requiring memory of object location. Whereas, the former experiments more consistently evoked activity on the bilateral posterior inferior frontal gyrus, the latter evoked more consistent activation on the posterior superior frontal gyrus bilaterally. In the present study, we investigated differences in task-based (MACM) and task-free (resting state) functional connectivity of these regions used as seeds. Across both states, the bilateral IPS showed significantly stronger connectivity with the ventral, while medial SPL and bilateral IPL showed significantly stronger connectivity with the dorsal seed. In a further step, we took these ensuing parietal areas as seed regions and investigated their functional connectivity using the same approach. The IPS then showed stronger connectivity with the bilateral ventral visual stream as well as with the seed on the bilateral posterior inferior frontal gyrus. The IPL/SPL on the other hand showed stronger connectivity with the bilateral dorsal visual stream and the seed on the bilateral posterior superior frontal gyrus. Our results demonstrate two largely segregated pathways of functional connectivity from the visual cortex via the parietal lobe to the posterior frontal cortex.

### Meta-analytic connectivity modelling versus resting-state correlations

In comparison to task-free resting state connectivity, task-based MACM delineates networks, which are concurrently recruited by an extended range of tasks and should therefore be able to reflect robust networks of coordinated activity in response to external task-demands. In contrast to the more prevailing operationalization of functional connectivity as coherent fluctuations in (resting-state) time-series, in MACM the unit of observation is thus represented by a particular neuroimaging experiment. Large-scale databases such as BrainMap have facilitated the assessment of such task-based functional connectivity analyses, which should present an optimal complement to resting-state functional connectivity analyses. Whereas the former delineates interactions during an externally driven, task-based state, the latter reveals interactions in an endogenously driven, task-free state (Eickhoff and Grefkes 2011).

It is important to appreciate that both approaches not only differ in the (presumed) confounds [task-based (MACM) functional connectivity is intrinsically constrained to tasks that may be performed in a scanner environment and influenced by spatial uncertainty inherent to neuroimaging results whereas task-free (resting state) functional connectivity may be more susceptible to preprocessing and physiological confounds]. Rather, they also represent different conceptual models: While MACM networks are externally controlled and targeted at stimuli and reactions, resting state networks reflect ongoing, endogenously controlled cognition.

Given this complementary nature, a combination of both approaches allows the delineation of functional connectivity independently of the current “mode” of brain function (externally structured or endogenously controlled). Thus, a robust estimation of the functional connectivity patterns of a particular seed region may consist of assessing task-based (MACM) as well as task-free (resting-state) functional connectivity and constraining inference to those regions showing convergent evidence in both approaches. It may be argued that convergent findings across both approaches, as presented in the current study, should represent the core of the respective network-interactions because the ensuing regions show the respective coupling pattern across two fundamentally different “modes” (Jakobs et al. 2012).

### Functional and anatomical connectivity

Functional connectivity as the temporal coincidence of spatially distant neurophysiologic events (Friston et al. 1996) represents a powerful technique for delineating functional interactions, but is fundamentally correlative and hence does not necessarily imply a causal relationship or indicate direct (anatomical) connections. The possibility to identify “real” anatomical connections between two neurons via direct unisynaptic axonal connections in turn is only provided by invasive tracer studies in animals such as non-human primates. It is thus important to remember the distinction between functional connectivity as an indicator of interacting networks and (invasively demonstrated) axonal connections. Whereas the latter show direct anatomical connections but are usually limited to monosynaptic interactions, the former, as investigated in our study, serves to characterize interacting nodes of brain networks. These, however, must not necessarily be directly linked to each other by an axonal connection. Open questions on between-species homology further complicate the comparison of (human) functional connectivity to anatomical connectivity in non-human primates. In particular, divergent findings may not only originate from differences in the employed methods and in conceptual differences between anatomical and functional connections but also from evolutionary changes between, e.g., macaque monkeys and humans (for a detailed discussion cf. Eickhoff et al. 2012, b; Tomassini et al. 2007).

Keeping the above limitations in mind, however, the delineated fronto-parietal connections seem to match rather closely with previous findings on the axonal connectivity of regions that are sited in similar locations of the monkey posterior frontal cortex (Luppino et al. 1999; Matelli et al. 1998). Most of these tracer studies revealed an association between primate posterior-inferior frontal cortex and the intraparietal sulcus (Godschalk et al. 1984; Kurata 1991; Luppino et al. 1999; Tanne-Gariepy et al. 2002). This frontoparietal circuit has been discussed to be involved in encoding peripersonal space coding for movements including grasping (Luppino et al. 1999; Matelli and Luppino 2001). In contrast to these connections, the posterior-superior frontal cortex was reported to show strong anatomical connections with the superior parietal lobule (Johnson et al. 1996; Matelli and Luppino 2001) as well as the medial intraparietal area MIP and adjacent inferior parietal lobule (Matelli et al. 1998). These connections seem to play a role in controlling (reaching) movements in space and higher order somatosensory elaboration (Matelli and Luppino 2001). In spite of the vastly different techniques (functional connectivity mapping versus invasive tracing of axonal connections), our current results thus align well with these observations, which in turn may be seen as providing further support for the observed distinction between the vPMC/IPS and dPMC/SPL circuits.

Moreover, anatomical connectivity of ventral and dorsal premotor cortex in humans has recently been investigated using tractography algorithms based on diffusion weighted imaging (Tomassini et al. 2007). The connectivity patterns revealed in that study are in generally close agreement with data from non-human primates as described above and the findings from the current study. In particular, Tomassini et al. (2007) found strong anatomical connectivity between the dorsal premotor cortex and the superior parietal lobule via the first branch of the superior longitudinal fasciculus (SLF I), a circuit which has been argued to play a crucial role for movements in space (Makris et al. 2005). The same study (Tomassini et al. 2007) also revealed anatomical connectivity between the ventral premotor cortex and the intraparietal sulcus along the third branch of the superior longitudinal fasciculus (SLF III), i.e., a pathway that has been associated with the encoding of grasping actions and hand shape (Koch et al. 2010; Makris et al. 2005).

Functional connectivity in the human brain has also been repeatedly investigated using ICA decomposition of resting-state fMRI. These investigations revealed various networks of coherent resting state fluctuations that are often in good congruence to task-based functional connectivity, i.e., co-activations (Laird et al. 2011b; Smith et al. 2009). In distinction to our findings of fronto-parietal-visual networks, these investigations often feature the ventral visual cortex as a distinct component not containing parietal or posterior frontal regions. The aforementioned studies (Laird et al. 2011b; Smith et al. 2009) did, however, reveal a network (labels as component #7 in these papers) that seems to correspond very well to the connectivity patterns observed for the posterior superior frontal gyrus in the present study. In turn, another component (#15) resembles the network identified as connected with the posterior inferior frontal gyrus. It may be noted, that throughout the resting-state ICA literature, similar components are often referred to as the dorsal and ventral attention network (Corbetta et al. 2008; Corbetta and Shulman 2002). While the dorsal attention network, the mentioned ICA component and the connectivity of our superior seed indeed show a high degree of similarity, a conspicuous difference emerges when comparing the current data to the ventral attention network as identified by task-based neuroimaging or ICA decomposition of resting state data. Whereas the ventral attention network has been described to specifically feature the right middle frontal gyrus as well as the right temporo-parietal junction in this hemisphere, these regions were not found to be connected to our seed on the posterior inferior frontal gyrus specific for memory of object identity.

In summary, our results thus provide converging evidence for segregated fronto-parietal pathways linking the vPMC to the intraparietal sulcus and the dPMC to the superior (and posterior inferior) parietal lobule. What is remarkably, though, is that we based our seed regions on a contrast of different working-memory tasks rather than any motor-related behaviour and still observed patterns of parietal connectivity very much like those known for action-related circuits. The convergence between the connectivity patterns of regions supporting memory for object identity and object location and those for ventral and dorsal premotor areas as well as the performed functional characterization (Fig. 7) hence provides evidence that the mentioned cognitive facilities are supported by networks also holding action-related functions.

### Functional roles of the posterior inferior and posterior superior frontal cortex

The hypothesis voiced in the conclusion to the previous section is corroborated by a comparison of the two seed regions with the functional definition of the vPMC and dPMC provided in a large-scale meta-analysis of motor control tasks (Mayka et al. 2006). In particular, the centre locations for the ventral and dorsal premotor cortex from the study of Mayka et al. (2006) are in good congruence with our present findings, underlining that the seeds obtained from an analysis of working-memory related activity may indeed reveal the ventral and dorsal premotor cortex as argued from their similar connectivity. Evidently, both vPMC and dPMC are associated with a large range of action-related tasks and behaviours. While the human ventral premotor cortex was implicated in grasp execution (Binkofski et al. 1999; Davare et al. 2006), predictive scaling of grip force (Dafotakis et al. 2008) and visually cued finger tapping (Ruspantini et al. 2011), the dorsal premotor cortex seems to be more involved into the remapping of arm position (Lee and van Donkelaar 2006) and the coupling between grasping and lifting objects (Davare et al. 2006). This is supported by human lesion studies, which showed that lesions in the dorsal premotor cortex clearly impair goal-directed actions (Candidi et al. 2008; Petrides and Pandya 2002). This short summary may illustrate the overarching supposition that ventral premotor areas are more tuned towards fine motor skills and in particular finger movements, whereas dorsal premotor areas are stronger involved in coarse movements in space (Davare et al. 2006; Rizzolatti et al. 2002). This anatomical functional distinction between ventral and dorsal aspects of the precentral gyrus is also reflected in the behavioural domain analysis as shown in Fig. 7. While the dorsal seed and the associated network were associated to action-related behavioural domains and spatial tasks, the ventral seeds and the connected network were more related to visual shape and configuration as well as in somatosensory and bodily perception.

In spite of ongoing discussions about the role of the premotor cortex in working memory, we would feel confident to argue that the locations differentially recruited by WM-related processes indeed correspond to the vPMC and dPMC, respectively. This view also resonates well with previous reports that the ventral premotor cortex plays a crucial role in non-spatial working memory tasks (Swartz et al. 1995) and together with the intraparietal sulcus stores information about manipulable objects (Mecklinger et al. 2002). The dorsal premotor cortex in turn has been already discussed in the context of spatial (nonverbal) auditory working memory tasks (Salmi et al. 2010). In summary, we would thus conclude that our seeds indeed reflect the ventral and dorsal premotor cortex in humans, with memory for object identity (non-spatial features) recruiting the ventral and memory for object location (spatial features) recruiting the dorsal premotor cortex.

### Connections to visual areas

We tested for parieto-occipital connections by performing a supplementary functional connectivity analysis, seeded from those parietal regions that showed differential connectivity to premotor areas engaged in “what” and “where” memory. We expected parietal regions connected to regions engaged by memorizing object identity, that is the IPS, to connect to areas of the ventral visual stream, in particular the fusiform gyrus (Orban et al. 2004). Such interactions were indeed present and, in particular in the fusiform gyrus, significantly stronger for those parietal regions connecting to the vPMC as well as for the vPMC itself (cf. Figs. 5a, 6a). The fusiform gyrus as part of the ventral extrastriate visual cortex in turn has strongly been implicated in object recognition, representation and processing (Eickhoff et al. 2007b; Grill-Spector and Malach 2004; Orban et al. 2004). Especially, there is convincing evidence for multiple domain specific “higher” visual areas in this region. Our data thus provide further evidence for the presence of an object centred processing stream connecting the cortex around the fusiform gyrus (Grill-Spector and Malach 2004), the anterior intraparietal sulcus (Grefkes et al. 2001) and the ventral premotor cortex. This stream seems to be involved in representing objects or their features both at an abstract-cognitive level (memory) as well as in the context of potential fine motor requirements for manipulation (action).

We did not find significantly stronger connectivity of the dorsal versus ventral seed within the (occipital) visual cortex (cf. Fig. 5a). When seeding from those parietal areas, however, stronger connectivity differences emerged in the lateral (area V5 and dorsally adjacent regions) and particularly in the medial (from V3d to the parieto-occipital sulcus) visual cortex (Fig. 6a). These regions are this significantly stronger connected to parietal regions interacting with the dorsal as opposed to those interacting with the ventral seed. The extended cluster in the medial visual cortex also included the superior parietal-occipital cortex (SPOC), which has been argued to correspond to the PRR (Cavina-Pratesi et al. 2010; Connolly et al. 2003; Fernandez-Ruiz et al. 2007). There is accumulating evidence, that this region is a key node in the cortical network for arm movements and particularly important for visuomotor transformations in the context of reaching (Batista et al. 1999; Snyder et al. 1997). Our functional connectivity analysis revealed that those parietal regions that are stronger connected to the dorsal seed, which is selective for memorizing object location, are in turn strongly coupled with a key region for coordinating reaching movements as well as those “lower” visual areas that provide the relevant input on object locations (Fig. 6a). The current analysis thus presents strong evidence for a location centred processing stream connecting (in particular) dorsomedial visual areas with the putative PRR, the superior and posterior inferior parietal lobule and the posterior superior frontal cortex. This stream seems to be involved in representing locations in space both at an abstract-cognitive level (memory) as well as in the context of potential reaching towards them (action).

Here it is important to note, that our analysis did not aim at identifying analogues to previously reported dorsal and ventral visual streams or even the classical dorsal/ventral stream proposed by Ungerleider and Haxby (1994). Rather, we here investigated whether there was a distinction in the functional connectivity with the visual cortex of those parietal areas that were differentially connected to the posterior inferior and posterior superior frontal cortex. That is, we investigated whether segregated pathways from frontal via parietal to visual cortex could be identified seeding from posterior frontal regions identified as relevant for “what” and “where” memory, respectively.

### Connections to prefrontal areas

Interestingly, we did not find a clear segregation of the LPFC in our analysis in spite of previous evidence towards such distinction (Rao et al. 1997; Wilson et al. 1993; Ungerleider et al. 1998). In particular, several previous studies have argued for an integration of the prefrontal cortex in distinct “what” and “where” streams. In human studies the inferior prefrontal cortex has been identified to play a role in working memory for object identity, while the superior prefrontal cortex seems to play a role in remembering object location (Ungerleider et al. 1998). Further studies showed differential coupling between the LPFC and premotor cortices during serial information processing and multiple-step cognitive manipulation (Abe et al. 2007; Abe and Hanakawa 2009). Moreover, non-human primate single cell recordings showed a clear segregation of the LPFC neurons into object recognition and spatial domains (Rao et al. 1997; Wilson et al. 1993). In our own analyses, however, only the caudal part of the lateral prefrontal cortex showed connectivity with both seed regions in the conjunction analysis (Fig. 5b). This coupling was moreover stronger with the (topographically closer) ventral seed (Fig. 5a). Given that the close proximity of this region with the vPMC may well explain its stronger connectivity with this region as opposed to the dPMC, we would thus be cautious to infer any prefrontal segregation from the current functional connectivity analyses.

In this context, it is important to point out, that we performed a voxelwise whole-brain analysis in order to test for regions, showing distinct functional connectivity with the seed regions, without any a priori hypotheses. The absence of a clear segregation of the LPFC may therefore not be attributed to negligence of this region but represents a true null-result, i.e., the prefrontal cortex was assessed as any other part of the brain but did not feature significant differences in functional connectivity to our seeds. Even though the organization of the prefrontal cortex still needs further investigation and conclusions therefore can only be speculative, we would propose that (at least this region of) the human LPFC is responsible for more abstract global processes, or in other words, occupies a more integrative role rather than being part of a “what” and “where” distinction.

### Cognition and motor behaviour: conclusion

It could be argued that the present study showing connectivity of the posterior inferior frontal gyrus with the IPS and ventral visual cortex as well as connectivity of the posterior superior frontal cortex with the IPL/SPL,and dorsal/medial visual cortex is primarily a replication of previous work on the organisation of premotor-parietal networks in humans and monkeys. While we were indeed able to show similar connections as previous studies on the premotor cortex, it is important to empathize that these findings were obtained when seeding from regions showing differential involvement in WM tasks. That is, premotor networks were replicated in spite of the fact that the seeds were defined by differential engagement in cognitive (WM) functions rather than motor behaviour. The current study thus showed that regions in the posterior frontal cortex preferentially engaged by memory for object location and identity, respectively, are part of similar networks as discussed for the ventral and dorsal premotor cortex. This strongly argues for a congruence between the seed regions defined by WM functions and the premotor cortex and thereby sheds an interesting light on the relation of cognitive and motor systems.

Our seeds were defined by assessing differential neural correlates of working memory for object identity or object location, that is, they were specified exclusively by their cognitive functions. Nevertheless, their location and parietal connectivity suggests a close homology to premotor areas in monkeys and humans (vPMC/dPMC). Functionally, this distinction in the primate premotor system has been related to differential involvement in reaching movements in space as opposed to fine motor skills for manipulation (for macaques see (Rizzolatti 1987); (Raos et al. 2003); for examples of a similar distinction between human premotor areas see (Dafotakis et al. 2008; Davare et al. 2006). A similar distinction with respect to action-related processes seems to hold for parietal areas differentially interconnected with these regions, whereas ensuing distinctions in the visual components of the delineated pathways pertain to more general location versus object centred processing. Thus, the differentiation of the visual components of the assessed pathways matches up very well with the cognitive distinction underlying the definition of the frontal seed regions. The seed defined by preferential recruitment in memory for object identity is connected to regions in the ventral visual cortex involved in object-processing and recognition. The one defined by memory for object location is connected to location-coding regions in the dorsal visual cortex. How to reconcile this segregation with the distinction of the same visual-parietal-premotor streams in motor actions, in particular reaching versus grasping?

We would argue that object (ventral, “what”) and location (dorsal, “where”) centred visual-parietal-premotor systems are shared by both cognitive and action-related processes and hence the same neural system may support memorizing object location in space and reaching towards it, while another system may underlie both memory for object properties and identity (like surface, colour or shape) and finger positioning for manipulation. In such scenario, the visual nodes would provide the analysis of the sensory input as well as first conceptual representations. Potentially, parietal areas may code supra-modal representations arising not only from visual but also auditory and somatosensory “what” and “where” information and at the same time tie (potential) motor actions towards these. Premotor regions would then most likely hold the most utilizable or output-ready representation, which may be used to perform an action—or judge whether the probe matches the previously memorized object in the relevant dimension. From an evolutionary perspective, it is well conceivable that these pathways were originally primarily devoted to action-related processing (though the distinction between cognition and action becomes blurred when it comes to simple mnestic functions present in virtually all mammals) and have assumed more abstract cognitive functions in primates and particularly humans. That is, the decisive step in the evolution of neural systems for action and cognition may be the decoupling of action execution from the preparatory aspects, using exactly the same brain systems to address abstract problems. Thus, many cognitive functions may be simulations of motor preparation after detaching the need for sensory input and movement output.
